# A Pilot Trial to Evaluate the Accuracy of a Novel Non-Invasive Glucose Meter

**DOI:** 10.3390/s21206704

**Published:** 2021-10-09

**Authors:** Yair Schwarz, Noa Konvalina, Amir Tirosh

**Affiliations:** 1 Sackler Faculty of Medicine, Tel-Aviv University, Tel-Aviv 6997801, Israel; Yair.Schwarz@sheba.health.gov.il; 2 Division of Endocrinology, Diabetes and Metabolism, Sheba Medical Center, Tel-Hashomer. Derech Sheba 2, Ramat-Gan 5266202, Israel; Noa.Konvalina@Sheba.health.gov.il

**Keywords:** glucometer, non-invasive glucose measurement, non-invasive glucose meter, radio frequency, self-monitoring of blood glucose

## Abstract

The non-invasive self-monitoring of blood glucose (SMBG) has been the subject of intense investigation over recent decades. We conducted a pilot study designed to examine a novel non-invasive glucometer, the HGR GWave, utilizing radiofrequency (RF) sensing. Blood glucose levels assessed by this HGR prototype were compared to measurements performed by a hexokinase core laboratory assay during an oral glucose tolerance test (oGTT) for 5 subjects with type 2 diabetes. The HGR glucose meter readings were also compared to two Abbot Freestyle^®^ glucose meters, which were also used for calibration. The accuracy of the results was evaluated through the calculation of relative absolute difference (RAD), specified percentage differences between 43 reference glucose measurements, and using comparator measurements. The median RAD was −4.787. We detected 79.04%, 92.99% and 97.64% of HGR readings within ±10%, ±15% and ±20% of the reference glucose measurements. The HGR readings had a high correlation with reference lab glucose measurements with R^2^ = 0.924 (95% CI 0.929–0.979; *p* < 0.0001). When compared to the Freestyle^®^ glucose meters 94.3% and 100% of the readings were within ±5% and ±10%, with R^2^ = 0.975 (0.975–0.994; *p* < 0.0001). The HGR prototype glucose meter was found to be accurate in detecting real-time blood glucose during an oGTT in this small pilot study. A study with a broader range of blood glucose levels is needed to further assess its accuracy and its suitability for clinical use.

## 1. Introduction

According to a report from 2018, the crude estimate for diabetes prevalence in the United States population is 10.5% (34.2 million people), with an estimated 26.8% of adults over the age of 65 years suffering from the disease. An additional 88 million adults live with prediabetes [1]. The projected prevalence of diabetes in 2050 is estimated to rise to 33% [2]. Self-monitoring of blood glucose (SMBG) is an integral component of therapy for patients with diabetes, with monitoring frequency differing according to diabetes type, patient therapy (insulin versus non-insulin) and patient characteristics. According to the American Diabetes Association (ADA) guidelines published in 2021, the recommended frequency of monitoring is up to 6–10 times per day [3]. Among children with type 1 diabetes each additional daily measurement correlates with a 0.4% reduction in hemoglobin A1c (HbA1c) [4], while <3.5 daily measurements correspond with poor glycemic control (HbA1c > 8%) [5]. Among patients with type 2 diabetes using insulin, more frequent SMBG was associated with improved glycemic control, especially in patients with uncontrolled diabetes at baseline [6]. While the frequency of SMBG and debates about its effectiveness in non-insulin treated patients with type 2 diabetes has caused controversy, it has been demonstrated that a structured SMBG program in insulin-naïve patients correlates with greater confidence in treatments and with improved glycemic control [7].

Currently, approved blood glucose meters, mostly relying on electrochemical technology, require a small amount of blood to be drawn by finger-pricking or the subcutaneous implantation of a sensor [8]. The burden of frequent SMBG was shown to be associated with mood disturbances, a lower diabetes-related quality of life, less recognition of SMBG importance and higher glycated hemoglobin [9]. Some of the barriers to SMBG are related to current techniques being used, and can include a fear of needles and pain and the inconvenience and the cost of test strips and lancets [10]. From a global, socioeconomic perspective, the use of frequent SMBG requires vast amounts of disposable medical supplies for which costs may at times equate to, or even surpass, the cost of diabetes medication regimens [11]. The use of flash glucose monitoring in type 1 diabetes patients may only marginally save costs when compared to SMBG, and potential savings depend on the frequency of SMBG and prevention of adverse events, such as hypoglycemia [12].

Non-invasive blood glucose monitoring devices are being rigorously researched as a method to overcome the drawbacks of conventional methods for SMBG [13]. Research over the past 40 years has evaluated multiple techniques to assess real-time blood glucose non-invasively, using methods such as bioimpedance, electromagnetic and fluorescence technologies, spectroscopy, and reverse iontophoresis, among others [14]. The possibility of using radio-frequency (RF) in sensing for blood glucose level characterization has also been investigated but has so far failed to mature as an alternative to invasive SMBG [15]. The current study aims to assess the accuracy of non-invasive glucose measurements in patients with diabetes using a real-time RF based technology.

## 2. Materials and Methods

### 2.1. The Device

**Figure 1:**
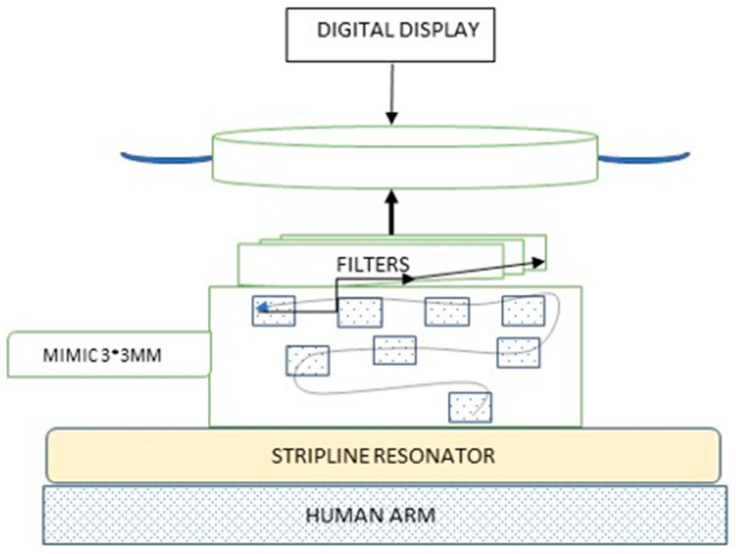
The HGR glucose meter design. The RF wave is generated in the patient arm. The return wave is cleared from all signals other than glucose by specialized filters. The uniqueness of the glucose meter lies in the dielectric plate and the filters.

HGR non-invasive glucose meter technology is based on the electrical reaction of biological tissue to the broadcasted RF wave, and the dielectric change in the tissue in correlation with the change of blood glucose levels (Figure 1).

The glucose meter utilizes an on-chip radio frequency generator with a frequency range between 19 Hz and 25 GHz. The reaction is measured on a dielectric material when the electromagnetic field flux is generated in a unique range. The dielectric material can be positioned at the cubital fossa of the patient’s arm, around the elbow or around the wrist. The emission level of the RF generator implanted in the glucometer is of a 1.48 specific absorption rate (SAR) in watts per kg (W/kg) and the device is powered using standard, commercially available batteries. The device provides the estimated blood glucose levels within seven seconds.

### 2.2. Study Participants

Five volunteers aged 18 years or older with type 2 diabetes and an HbA1c between 6.0–8.0% (42–64 mmol/mol), regardless of treatment, were included in the study. Pregnant women were excluded from the study.

### 2.3. Study Location

The Clinical Research Unit of the Division of Endocrinology, Diabetes and Metabolism at Sheba Medical Center, Ramat-Gan, Israel.

### 2.4. Study Procedure Overview

A 75 g oral glucose tolerance test (oGTT) was conducted. Blood glucose levels were measured at pre-defined time points (detailed below) simultaneously by the HGR glucose meter, and at the Sheba Medical Center core laboratory, which is an ISO15189 certified laboratory. Blood samples for glucose measurements were collected in designated tubes containing a separating gel and were centrifuged within 1 min of their collection on site to minimize in-vitro glycolysis. Following their centrifugation and using pneumatic tube transport, test tubes were sent to the core laboratory for immediate analysis using the hexokinase assay (AU5800, Beckman Coulter). The tests performed at the core laboratory were defined as reference glucose levels. In addition, blood glucose levels were simultaneously measured using two point of care hand-held glucose meters (Abbot FreeStyle^®^). The blinding of the glucose reference levels was achieved by measuring and recording the HGR readings, prior to measuring them with FreeStyle^®^ and receiving the core laboratory results.

### 2.5. Study Procedure Step by Step

1. Patients reported at 8:00 a.m. after an 8 h fast (no caloric intake, water permitted). A peripheral intravenous catheter was placed in the antecubital fossa. Baseline blood glucose was collected and sent for analysis at the core laboratory.
2. The HGR glucose meter was calibrated at the beginning of the day, before initiating any study procedures using Abbot FreeStyle^®^ glucose meters and standard solutions.
3. Blood glucose was measured non-invasively using the HGR device by placing the reading plate of the device on the anterior part of the wrist. The capillary blood glucose of study participants 2–5 was recorded at the baseline and at all the time points, using two Abbot FreeStyle^®^ glucose meters.
4. Study participants consumed 75 g of glucose (standard solution for an oGTT made by adding 75 g glucose [FLORIS, Industrial Park Misgav, Israel] to 180 mL water) within 15 min.
5. Additional sets of measurements (venous blood, non-invasive reading and capillary blood) were taken at 15, 30, 45, and 60 min following glucose ingestion.
6. Between 60 and 180 min samples were collected every 30 min, with a total of 9 sets of measurements per subject from 0 to 180 min.

### 2.6. Study Outcomes

Study outcomes included:

1. The median related absolute difference (RAD) between HGR glucose measurements and reference glucose levels. RAD was calculated using the following formula:
  RAD = (*Obg_i_* − *Rbg_i_*)/*Rbg_i_*
  *Obg_i_* is the *i*th HGR estimated glucose
  *Rbg_i_* is the *i*th reference blood glucose
2. The percentage of the readings within ±5%, ±10%, ±15% and ±20% of the reference readings were calculated.
3. The Pearson correlation between HGR glucose meter readings and reference glucose values was calculated using GraphPad Prism V.6.

## 3. Results

Five subjects (3 males and 2 females) were included in the study. The mean age of participants was 59.4 years. The mean fasting glucose level was 116.6 ± 7.4 mg/dL (mean; SD) and the mean HbA1c was 7.0% (53 mmol/mol).

A sum of 43 values of glucose measurements were recorded in the core laboratory (expected number 45) with 2 missing values–subject 2 at time 30′ and subject 4 at time 15′.

The mean glucose during the oGTT was 182.2 ± 57.22 mg/dL (mean; SD) ranging between 73.0 mg/dL and 299.0 mg/dL.

The median RAD was −4.787 mg/dL with ranges between −23.08 and 8.209 mg/dL. The 25% percentile was −11.59 mg/dL, and the 75% percentile was 2.564 mg/dL (Table 1).

**Figure 2:**
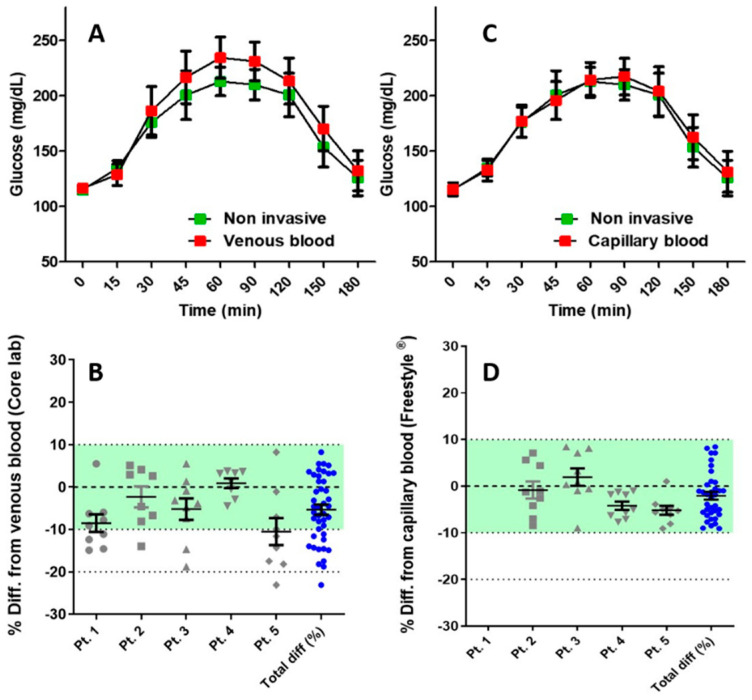
(**A**) Comparison of oral glucose tolerance test (oGTT) results between capillary blood (red) and HGR glucose readings (green). (**B**) Percentage difference of HGR glucose readings versus glucose levels of venous blood measured by the reference laboratory for each subject, and the full cohort. (**C**) Comparison of oGTT results: Abbott FreeStyle^®^ (red) and HGR (green). (**D**) Percentage difference of HGR readings versus capillary blood readings by Abbott FreeStyle^®^ glucose meters for each subject and the full cohort.

Of all HGR glucometer readings, 79.04% were within ±10% percent of reference glucose values, 92.99% were within ±15% percent of reference glucose values and 97.64% were within ±20% of reference glucose values (Table 2 and Figure 2A). The percentage of differences are presented graphically for each subject (Figure 2B), with subjects 1 and 5 having a tendency for lower and more scattered percentage differences compared to subjects 2, 3 and 4.

**Figure 3:**
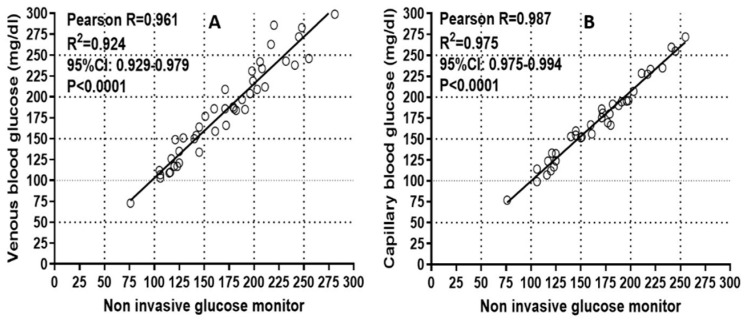
(**A**) Pearson correlation between HGR glucose meter readings and venous blood glucose readings by reference lab. (**B**) Pearson correlation between HGR glucose meter readings and capillary blood glucose readings by Abbott FreeStyle^®^ glucose meters.

The Pearson correlation (Figure 3A) between HGR readings and core lab values was calculated using R^2^ of 0.924 (95% CI 0.929–0.979; *p* < 0.0001). The Pearson correlation (Figure 3B) between HGR and Abbott FreeStyle^®^ glucose meter readings was R^2^ = 0.975 (95% CI 0.975–0.994; *p* < 0.0001). Further, 94.3% and 100% of the HGR glucose meter readings were within ±5% and ±10% of the of the capillary blood glucose measurements performed with Abbott FreeStyle^®^ glucose meters (Table 2 and Figure 2C). The percentage of difference is presented graphically for each subject (Figure 2D).

## 4. Discussion

This first in-human pilot study was designed to suggest the possible utility of the HGR glucose meter as a non-invasive device for monitoring glucose levels in patients with diabetes. The analysis performed in our study was based on the U.S. Food and Drug Administration (FDA) guidelines published in September 2020. Based on the guidelines, it must be demonstrated that across the prospective range of a device, glucose levels measured by the proposed devices are within ±15% of the reference, 95% of the time and within ±20% of the reference 99% of the time [16]. In our pilot study, albeit being limited to 43 readings and being insufficiently powered to satisfy FDA criteria, HGR glucose meters achieved 92.99% of readings within ±15% of the comparator results and 97.64% within ±20% of the comparator results. These results need to be validated in a larger cohort of patients and with a broader range of blood glucose levels. Nevertheless, this pilot study demonstrates the potential of using this non-invasive RF glucose meter in patients with diabetes, at least in the range of 70–300 mg/dL achieved in this study.

The technology of RF and microwaves, for non-invasive glucose monitoring has been well-researched; research includes in-vitro studies on solutions using various methods, and a limited number of in vivo studies, [15,17,18]. In one study, a pair of patch antennas operating at 60 GHz were tested on ten healthy, male volunteers who underwent an intravenous glucose tolerance test (IVGTT). The results of the study showed a good correlation in two of the subjects, albeit with a time lag, while in the other eight subjects, the data collected demonstrated distortions, possibly due to hand motion and a sliding of the antennas during the test [17]. A proof of concept study using a microwave sensor integrated with low-cost radar in a healthy male volunteer presented a good correlation with invasive blood glucose monitoring by a standard glucose meter [18]. A third study was performed on an anesthetized pig, using a pair of opposing patch antennas as a sensor alongside an IVGTT. In this study, the sensor demonstrated a spike with a 13 min delay from the point of the glucose spike [19]. Our study differs from studies employing RF systems published to date, in that it includes participants with diabetes, employs a well-defined oGTT with laboratory based reference values, and demonstrates a high degree of RF device accuracy in all studied subjects.

There are numerous challenges for the employment of RF technology to the non-invasive monitoring of blood glucose, including the need for high sensitivity due to very small changes in the permittivity and conductivity of the plasma caused by changing glucose levels. Other challenges are attributed to “real life” factors which can affect dielectric properties, such as skin temperature change, perfusion, sensor positioning and patient motion. Additional factors include the blood level of different gases and chemicals (including drugs) and differing tissue thickness between subjects [15]. Due to this, it should be noted that, in a study investigating the most popular 18 glucose meters in the U.S market, only six of the systems met the predetermined accuracy standard in all three tests [20]. Moreover, conventional electrochemical methods used in invasive glucose meters are potentially sensitive to technical errors of operation, as well as changes in blood components. These components include oxygen saturation and the interference of substances such as acetaminophen, uric acid and ascorbic acid [3], among others. Currently, only a small number of non-invasive glucose meters are available to patients, and some systems have been withdrawn from the market due to inadequate accuracy levels [13].

As a pilot study, our study has limitations that affect the generalizability of our results, such as the small number of patients. Another limitation derives from the controlled conditions of the study, which prevented us from assessing the performance of the HGR glucose meter during various real-life potential interferences, such as physical activity or changes in temperature.

## 5. Conclusions

In this pilot study, the RF based HGR glucose meter readings were compared to a laboratory hexokinase assay and commercially available glucose meter using RAD, prespecified percentage differences and correlations as outcomes. The HGR glucometer proved to be a potential non-invasive alternative method for point of care or continuous self-monitoring of blood glucose. Further research is needed to prove the adequate accuracy and precision of this novel methodology for glucose measurements across a greater population of people with diabetes as well as at a broader range of glucose levels. More specifically, its precision and accuracy need to be evaluated for glucose values lower than 70 mg/dL, to include values in both level 1 and level 2 hypoglycemia. In addition, testing its ability to detect values above 300 mg/dL, as well as rapid changes in blood glucose are also critical for its further development towards clinical use. Although our study compared the HGR glucometer to blood glucose measurements for pre-defined time points, it has the potential to be used as a real-time continuous glucose monitor, and it should be studied for this usage in future studies. We did not detect any signals of reading interference, but a careful assessment of the effects of medications, supplements, or other environmental exposures on the potential accuracy of the HGR glucose meter is needed in larger studies. In summary, the development of an accurate and reliable non-invasive glucose meter (for SMBG or CGM use) will likely confer a significant change in current diabetes management, resulting in better adherence to monitoring and an achievement of glycemic goals, as well as improved patient reported outcomes and higher satisfaction.

## Figures and Tables

**Table 1:** Relative absolute difference between HGR glucose meter and relative core lab glucose values.

Minimum	−23.08
25% Percentile	−11.59
Median Relative Absolute Difference	−4.787
75% Percentile	2.564
Maximum	8.209
Mean Relative Absolute Difference	−5.339
Std. Deviation	7.908
Std. Error	1.206

**Table 2:** Summary of data within specified percent difference from reference glucose values and capillary glucose values.

	Within +/− 5%	Within +/− 10%	Within +/− 15%	Within +/− 20%
Percent of glucose readings from reference glucose values	60.45%	79.04%	92.99%	97.64%
Percent of glucose readings from capillary glucose values	94.3%	100%	----	----

## References

[1] DHHS National Diabetes Statistics Report, 2020. https://www.cdc.gov/diabetes/library/features/diabetes-stat-report.html.

[2] American Diabetes Association (2018). Economic Costs of Diabetes in the U.S. in 2017. Diabetes Care.

[3] American Diabetes Association (2021). 7. Diabetes Technology: Standards of Medical Care in Diabetes—2021. Diabetes Care.

[4] Haller M.J., Stalvey M.S., Silverstein J.H. (2004). Predictors of control of diabetes: Monitoring may be the key. J. Pediatr..

[5] Murata T., Tsuzaki K., Yoshioka F., Okada H., Kishi J., Yamada K., Sakane N. (2015). The relationship between the frequency of self-monitoring of blood glucose and glycemic control in patients with type 1 diabetes mellitus on continuous subcutaneous insulin infusion or on multiple daily injections. J. Diabetes Investig..

[6] Machry R.V., Rados D.V., de Gregório G.R., Rodrigues T.C. (2018). Self-monitoring blood glucose improves glycemic control in type 2 diabetes without intensive treatment: A systematic review and meta-analysis. Diabetes Res. Clin. Pr..

[7] Fisher L., Polonsky W.H., Parkin C.G., Jelsovsky Z., Petersen B., Wagner R.S. (2012). The impact of structured blood glucose testing on attitudes toward self-management among poorly controlled, insulin-naïve patients with type 2 diabetes. Diabetes Res. Clin. Pr..

[8] Gonzales W.V., Mobashsher A.T., Abbosh A. (2019). The Progress of Glucose Monitoring—A Review of Invasive to Minimally and Non-Invasive Techniques, Devices and Sensors. Sensors.

[9] Tanaka N., Yabe D., Murotani K., Ueno S., Kuwata H., Hamamoto Y., Kurose T., Takahashi N., Akashi T., Matsuoka T. (2018). Mental distress and health-related quality of life among type 1 and type 2 diabetes patients using self-monitoring of blood glucose: A cross-sectional questionnaire study in Japan. J. Diabetes Investig..

[10] Chua S.-S., Ong W.M., Ng C.J. (2014). Barriers and facilitators to self-monitoring of blood glucose in people with type 2 diabetes using insulin: A qualitative study. Patient Preference Adherence.

[11] Klatman E.L., Jenkins A., Ahmedani M.Y., Ogle G.D. (2019). Blood glucose meters and test strips: Global market and challenges to access in low-resource settings. Lancet Diabetes Endocrinol..

[12] Hellmund R., Weitgasser R., Blissett D. (2018). Cost calculation for a flash glucose monitoring system for UK adults with type 1 diabetes mellitus receiving intensive insulin treatment. Diabetes Res. Clin. Pr..

[13] Bolla A.S., Priefer R. (2020). Blood glucose monitoring- an overview of current and future non-invasive devices. Diabetes Metab. Syndr. Clin. Res. Rev..

[14] Chung J.W., So C.-F., Choi K.-S., Wong T.K. (2012). Recent advances in noninvasive glucose monitoring. Med. Devices Evid. Res..

[15] Yilmaz T., Foster R., Hao Y. (2019). Radio-Frequency and Microwave Techniques for Non-Invasive Measurement of Blood Glucose Levels. Diagnostics.

[16] U.S Food and Drug Administration (2020). Self-Monitoring Blood Glucose Test Systems for Over-the-Counter Use Guidance for Industry and Food and drug administration staff.

[17] Saha S., Cano-Garcia H., Sotiriou I., Lipscombe O., Gouzouasis I., Koutsoupidou M., Palikaras G., Mackenzie R., Reeve T., Kosmas P. (2017). A Glucose Sensing System Based on Transmission Measurements at Millimetre Waves using Micro strip Patch Antennas. Sci. Rep..

[18] Omer A.E., Shaker G., Safavi-Naeini S., Kokabi H., Alquié G., Deshours F., Shubair R.M. (2020). Low-cost portable microwave sensor for non-invasive monitoring of blood glucose level: Novel design utilizing a four-cell CSRR hexagonal configuration. Sci. Rep..

[19] Cano-Garcia H., Saha S., Sotiriou I., Kosmas P., Gouzouasis I., Kallos E. (2018). Millimeter-Wave Sensing of Diabetes-Relevant Glucose Concentration Changes in Pigs. J. Infrared Millimeter, Terahertz Waves.

[20] Klonoff D.C., Parkes J.L., Kovatchev B.P., Kerr D., Bevier W.C., Brazg R.L., Christiansen M., Bailey T.S., Nichols J.H., Kohn M.A. (2018). Investigation of the Accuracy of 18 Marketed Blood Glucose Monitors. Diabetes Care.

